# Exploring the overuse of non-sterile gloves in operating theatres: a cross-sectional survey and interview study

**DOI:** 10.1136/bmjopen-2025-102835

**Published:** 2026-06-02

**Authors:** Carys Batcup, Aws Almukhtar, Aarya Menon, Daniel Leff, Gaby Judah, Pelin Demirel, Talya Porat

**Affiliations:** 1 Dyson School of Design Engineering, Imperial College London, London, UK; 2 Faculty of Social and Behavioural Sciences, University of Amsterdam, Amsterdam, Netherlands; 3 Department of General Surgery, St Mary’s Hospital, Imperial College Healthcare NHS Trust, London, UK; 4 Department of Surgery and Cancer, St Mary’s Hospital, Imperial College Healthcare NHS Trust, London, UK

**Keywords:** Safety, Behavior, Hospitals, QUALITATIVE RESEARCH, SURGERY, Surveys and Questionnaires

## Abstract

**Abstract:**

**Objectives:**

To identify factors influencing unnecessary non-sterile glove use in operating theatres and to estimate how common these factors are across the UK.

**Design:**

Mixed-methods study using interviews and a cross-sectional survey.

**Setting:**

Imperial College Healthcare Trust for interviews and nationally across the UK for the survey.

**Participants:**

19 interviewees and 329 survey respondents, all clinical staff working in UK operating theatres.

**Outcome measures:**

Barriers and facilitators to unnecessary non-sterile glove use in operating theatres.

**Results:**

The findings highlight a combination of key drivers leading to the unnecessary use of non-sterile gloves: (1) lack of prioritisation of sustainability, (2) fears around negative patient outcomes, (3) strong social influences such as norms to use gloves, (4) the absence of clear guidelines and limited training on glove use, (5) availability of alternatives and quality of gloves and (6) beliefs about personal safety and habitual glove use. Respondents also suggested potential intervention strategies.

**Conclusions:**

67% of participants reported using gloves unnecessarily. Our findings highlight the role of habitual behaviour, social influences and unclear guidelines in driving this practice. Interventions should address these factors, for example, by clearly communicating when gloves should and should not be worn, encouraging changes to local social norms towards waste reduction, improving access to hand gel and supporting habit change to reduce unnecessary glove use and associated environmental impact.

**STRENGTHS AND LIMITATIONS OF THIS STUDY**

- The use of a mixed methods approach strengthens the findings through triangulation of qualitative and quantitative data.
- Use of the Theoretical Domains Framework, an established model of behaviour change, supports translation of these findings into the design of interventions to reduce non-sterile glove use.
- Despite efforts to recruit a diverse sample of interviewees and survey respondents, the findings may primarily represent the views of healthcare workers in England.
- Participants were more likely to be those interested in environmental change, possibly making them more motivated to engage in relevant research and behavioural changes compared with others in their field.

## Introduction

 Healthcare is a major contributor to climate change, responsible for at least 4% of global emissions.^1^ A single operation can generate up to 505.1 kg of carbon dioxide equivalents (CO2e), with single-use items being one of the main contributors,^2^ threatening two areas within the Planetary Boundaries Framework, namely Novel Entities and Climate Change.^3^ Operating theatres (OTs) produce up to 33% of hospital waste,^4^ therefore waste reduction and management is crucial to reduce their environmental impact. The most effective way to decrease CO2e of disposable items will always be to reduce use where possible, as opposed to reusing or recycling existing supplies.^5^

A key area for reducing the environmental impact of these consumable and disposable items is decreasing the use of single-use non-sterile gloves (NSGs).^6^ Approximately 3.6 billion NSGs are used every year within healthcare in England, especially in OTs,^7 8^ and this high usage results in NSGs being a major contributor to the environmental impact of healthcare.^9^ NSGs are recommended when healthcare workers are exposed to blood or bodily fluids, and in this case are essential for reducing the risk of spreading infections and therefore constitute appropriate use.^10 11^ However, gloves are often used unnecessarily (eg, when using whiteboards or computers): studies report that in up to 59% of instances NSG use is unnecessary and an average of six to seven gloves may be used inappropriately per operation.^12^,^15^ Unnecessary NSG use is associated with increased cross-contamination between patients, reduced hand hygiene compliance and microbial contamination from glove boxes themselves.^15^,^19^ Furthermore, NSG production is associated with severe human rights abuses.^20^

Reducing unnecessary use of NSGs requires changes in healthcare practitioners’ behaviour. While clinicians are often motivated to reduce their environmental impact, behaviour change tends to be slow.^5 21 22^ Most existing studies, which focus on reducing glove use for hygiene improvements, have identified barriers such as emotions (comfort, disgust) and habits.^1223^,^25^ However, there is a lack of research investigating barriers to reducing glove use in the specific context of OTs. Better understanding the influences of behaviour change is key to designing interventions that are both practical and effective.

Furthermore, previous studies on reducing glove use have not applied a behavioural framework to identify barriers and facilitators systematically and support more effective intervention design. In this study, we applied the Theoretical Domains Framework (TDF),^26^ which is designed to comprehensively cover potential behavioural influences and is frequently applied to healthcare behaviour and sustainability contexts.^22 27 28^ The TDF is also part of a wider approach to behaviour change, the Behaviour Change Wheel,^29^ which facilitates translating findings on behavioural influences into actionable intervention design.

Our aim was to explore influences on unnecessary use of NSGs in OTs. Given the interplay of theatre staff behaviour, social and organisational influences, we used a mixed-methods research strategy of interviews and an exploratory survey. We aimed to leverage the strengths of qualitative methods and a quantitative survey in order to gain a more holistic and deeper understanding of healthcare workers’ perceptions of NSG use, using the TDF to guide both methodologies to ensure theoretical consistency.^30 31^

## Methods

**Figure 1:**
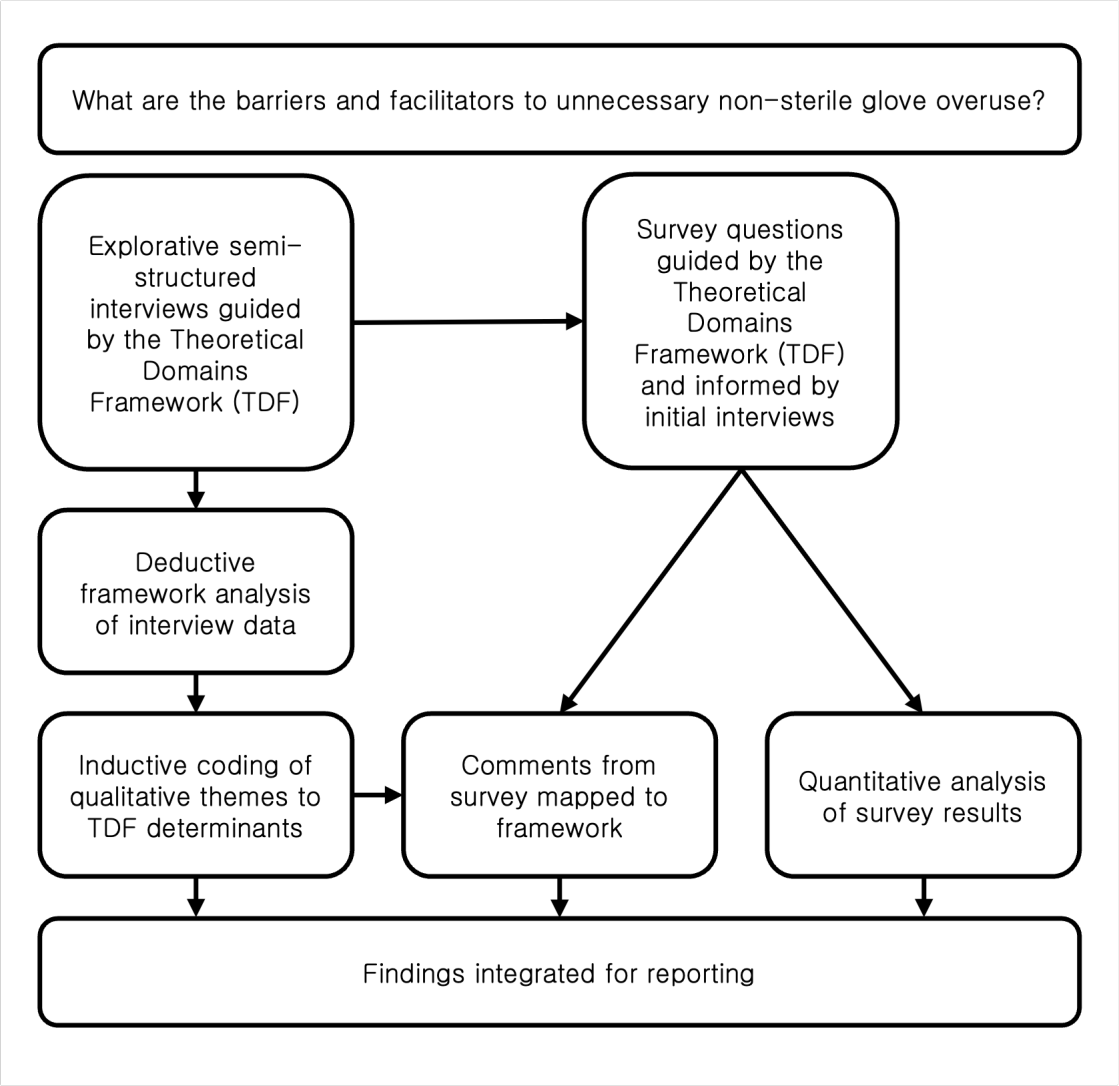
Logic diagram of methodological process.

The methodological process is summarised as a logic diagram in figure 1 for clarity.

### Interviews

#### Participants and design

Semi-structured interviews with staff working in OTs at Imperial College Healthcare NHS Trust hospitals. Purposive sampling was conducted to ensure recruitment from a variety of professional roles and levels of seniority. Predefined sampling targets were: ≥5 surgeons, ≥5 anaesthetists, ≥5 nurses and at least one operating department practitioner (ODP) to capture diverse perspectives on perioperative practice. Participants were recruited through internal networks and posters placed in hospitals. Interviews were conducted between 7 June 2023 and 7 September 2023, either online or in-person according to participant preference. The interview guide included general questions about participants’ role in the OT, followed by questions based on the TDF domains (see online supplemental file 1).

Interviews were conducted by two researchers: CB (female, MSc, a behavioural scientist experienced in qualitative research in health and sustainability) and AA (male, MBBS, MSc, MRCSEd, MFSTEd, a practising surgeon with sustainability expertise). AA had prior professional acquaintance with a small number of participants; no other prior relationships existed. Participants were informed that the study examined barriers and facilitators to green surgery practices and that interviewers were researchers from Imperial College London. Interviews were audio-recorded, transcribed and anonymised.

#### Analysis

Framework analysis^32^ was used to analyse interview data. First, two transcripts were coded inductively by two researchers (CB, AM), and ideas for themes were discussed. A further two transcripts were then coded independently, and again the codes and preliminary themes were considered. These initial themes were then discussed with the wider research team (AA, GJ, TP, PD), resulting in a coding framework. CB coded the remaining transcripts and created a framework of themes, sub-themes and relevant quotes. These were discussed by the research team before the final themes were decided on. Sub-themes were then deductively mapped to TDF domains.

### Survey

#### Participants and design

The national survey was delivered on the online platform Qualtrics. Participants were recruited through open, voluntary invitations disseminated via professional organisations (the Royal Colleges of Surgeons, Nursing and Anaesthetists), hospital posters and researchers’ networks. Formal response rates and probability sampling were therefore not possible, as the total number of potential participants was not obtainable. Inclusion criteria included participants aged 18 years or older, able to read and understand English, and currently working in an OT in the UK. Survey completion was incentivised through entry into a prize draw for a £100 voucher. Recruitment took place between 27 July 2023 and 15 April 2024.

Survey items are shown in online supplemental file 2, including items on professional role and seniority, frequency of unnecessary glove use, and perceived influences on glove use. Questions on influences were developed based on initial themes identified from the interviews, and encompassed all TDF domains. Greater emphasis was given to the domains identified as most relevant or important from interview findings. For TDF domains discussed in interviews, responses were captured on a 5-point Likert scale (1=strongly disagree to 5=strongly agree). For domains not discussed in interviews, participants indicated whether a particular influence was relevant to them (binary response). This approach was taken to reduce survey length and participant burden. Disgust was measured using items from a six-factor hygiene disgust scale (1=no disgust and 7=extreme disgust).^33^ A free text item was also included: ‘Do you have any other comments on reducing NSG use, e.g. whether this is possible or how it may be encouraged?’.

Items were developed in consultation with the hospital infection control team and the survey was piloted with seven OT staff (two anaesthetists, three surgeons, one theatre nurse and one ODP) to assess content validity, clarity and usability, and was revised to ensure role-specific relevance and minimise participant burden. Nurses answered the largest number of glove-use and influence items because they most frequently use NSGs in OTs. Surgeons and anaesthetists answered a subset of items relevant to their routine NSG tasks.

For all roles (nurses, surgeons, anaesthetists), unnecessary glove use was determined through the question asked to all groups: ‘using gloves to move a patient where there is no visible soiling’. This behaviour was chosen as all groups perform this action, and an infection control representative was consulted to ensure that gloves were not needed to perform this safely. Unnecessary glove use was treated as a categorical variable, with answers of ‘never’/‘rarely’ categorised as ‘appropriate glove use’, and answers ‘sometimes’/‘often’/‘always’ categorised as ‘unnecessary glove use’.

#### Data analysis

For the survey, descriptive statistics were calculated for all participant characteristics and outcome measures using JASP statistics software.^34^ The threshold for statistical significance was p<0.05. Multiple items measuring the same TDF domain were combined if Cronbach’s α was over 0.6, indicating good internal consistency. The binary measure of unnecessary glove behaviour was compared with the continuous behavioural influences using t-tests. Statistical analyses were not conducted on the binary behavioural influences on glove use due to low numbers, therefore absolute numbers are presented. Qualitative analysis of the open question was performed by coding comments to themes from interview data analysis, and any comments which did not integrate with this were coded into separate themes.

### Patient and public involvement

Patients and the public were not involved in this study, which focused on glove-use practices and behavioural influences among OT staff. Future intervention development and evaluation will incorporate patient and public involvement.

## Results

The interview and survey findings are presented together through an integrated thematic framework. Key themes initially generated from the qualitative analysis of interviews structure the results. All data were mapped onto each theme for a comprehensive view of the behavioural determinants influencing glove overuse. This technique aligns with established mixed-methods research principles, in which qualitative and quantitative data are analysed separately but integrated during interpretation.^35^

### Participant characteristics

19 participants participated in the interviews (see table 1 for participant characteristics). Interviews lasted between 22 and 64 min (mean 41 min). No participants declined participation or withdrew during the study. Thematic saturation was reached after 15 interviews, with the remaining interviews confirming saturation across roles.

**Table 1:** Participant characteristics for Phase 1 interviews; n=19

Characteristic	N (%)
Gender	
Male	9 (47)
Female	10 (53)
Role in operating theatres	
Nurse	6 (32)
Junior	2 (11)
Senior/theatre manager	4 (21)
ODP	1 (5)
Surgeon	8 (42)
Junior (SHO or below)	2 (11)
Intermediate (registrar)	3 (16)
Senior (consultant)	3 (16)
Anaesthetist	4 (21)
Senior (consultant)	4 (21)

ODP, operating department practitioner; SHO, Senior House Officer.

A total of 494 participants began the survey, of whom 431 completed it (87%). Of these 431, 102 participants were screened out (24%): 35 did not consent, 34 indicated that they do not work in an OT in the NHS and 33 selected ‘other’ as their job role (ie, not a surgeon, nurse, ODP or anaesthetist). Therefore, 329 participants (78%) were included in the analysis. The median time to complete the survey was 12.8 min.

Sample characteristics from the survey are in online supplemental file 3). 45% of the sample were nurses and ODPs, 21% were surgeons and 34% anaesthetists. Participants were from a range of seniority and experience levels. Most (45%) were aged 30–39; however, age ranges from 18 to 29 up to 60–69 were represented. 54% were female and 45% were male. 68% worked at a tertiary hospital, and the most represented specialties were emergency (39%), general (39%) and trauma (33%). 91% of participants worked in England, and of those 63% were based in London. There was a spread of experience in OTs, although most had 1–5 years of experience (33%). Free-text responses were provided by 145 (42%) respondents: 47 nurses and ODPs, 25 surgeons and 73 anaesthetists.

### Themes

Qualitative framework analysis from interviews resulted in six themes: 1. Sustainability is not prioritised in the organisation, 2. Patient outcomes are the absolute priority in OTs, 3. Social dynamics within the OT play a role in increasing waste, 4. Lack of availability or poor quality of facilities increases waste, 5. The importance of clear guidelines and training in NSG use reduction and 6. Individuals’ preferences and habits. A seventh theme was found in survey comments: participants’ suggestions for interventions to reduce glove use. These themes encompassed 17 sub-themes and 10 of the 14 TDF domains—goals, beliefs about consequences, emotion, environmental context and resources, knowledge, memory attention and decision processes, social influences, social/professional role and identity, optimism and reinforcement. The relations between the themes, subthemes and TDF domains are detailed in table 2.

**Table 2:** Themes, sub-themes and related domains from the Theoretical Domains Framework (TDF)

Theme	Sub-theme	TDF domain	Barrier/facilitator
Sustainability is not prioritised in the organisation	Other priorities in the NHS	Environmental context and resources, optimism	Barrier
	Culture of waste in the NHS	Environmental context and resources	Barrier
	Many individuals also have other priorities than sustainability	Goals	Barrier
	Individuals do not have motivation or incentivisation to reduce waste	Reinforcement, beliefs about consequences	Barrier
Patient outcomes are the absolute priority in operating theatres	Different surgeries’ requirements can lead to more waste	Memory, attention and decision processes, beliefs about consequences, environmental context and resources	Barrier
	Fear of something going wrong in surgery (for the patient)	Emotion, memory attention and decision processes, beliefs about consequences	Barrier
Social dynamics within the operating theatre can play a role in waste	Some differences as to who is 'in charge’, but often descriptions of being ‘told off’, etc	Social influences	Barrier
	Having a supportive or unsupportive team influences the amount of waste	Social influences	Barrier or facilitator
	Communicating clearly with the team, including nurses anticipating needs of the surgeon and therefore putting on gloves early	Social influences	Barrier or facilitator
	Perceived pressure to wear gloves due to social norms	Social influences	Barrier
Availability and quality of facilities	Glove packaging lends itself to waste, and gloves themselves can be poor quality	Environmental context and resources	Barrier
	Alternatives to gloves (ie, handwashing or hand sanitiser) are often not obvious or unavailable. However, gloves are readily available	Environmental context and resources, memory attention and decision processes	Barrier
The importance of clear guidelines and training in NSG use reduction	No known or clear guidelines for glove use	Knowledge	Barrier
	No standardised training for glove use	Social influences	Barrier or facilitator
Individuals’ preferences and habits	Habitual behaviour	Memory attention and decision processes	Barrier
	Identity of someone who is environmentally conscious	Social/ professional role and identity	Barrier or facilitator
	Fear of infection (exacerbated by COVID-19) and 'better safe than sorry’ approach to personal safety	Emotion, memory attention and decision processes, beliefs about consequences	Barrier
	Those with more years of experience or higher seniority tend to feel more comfortable and less worried about doing things ‘correctly’ or strictly following infection control practices	Memory attention and decision processes, beliefs about consequences, social/professional role and identity	Barrier or facilitator
Suggestions for interventions	Current campaigns or efforts to reduce glove use	/	/
	Environmental champion or people advocating for this	/	/
	Prompts, eg, glove counter or posters	/	/
	Education and training on impact of glove use and when and where to use	/	/
	Access to alternatives	/	/
	Ideas for other sustainability issues	/	/

NHS, National Health Service; NSG, non-sterile glove.

In the survey, 67% of participants reported using gloves unnecessarily while moving a patient when there is no visible soiling (ie, sometimes, often or always doing so). Quantitative results are described within the relevant themes and are summarised in tables3  4.

**Table 3:** Influences on unnecessary glove use (continuous response disagree^1^ –agree^5^)

Influences on non-sterile glove use with responses along a Likert scale:	TDF or other domain	Mean agreement for those who use gloves appropriately (SD; N)	Mean agreement for those who use gloves unnecessarily (SD; N)	t value (p)
I only use gloves when it is necessary to do so	Beliefs about behaviour	4.53 (0.89; 95)	3.84 (1.28; 210)	−4.73 (<0.001)*
When I wear non-sterile gloves in operating theatres, it is usually to protect the patient; wearing fewer non-sterile gloves in operating theatres would increase the risk of infection for the patient(Cronbach’s α = 686)	Beliefs about consequences	2.25 (1.13; 56)	3.09 (1.08; 137)	4.82 (<0.001)*
When I wear non-sterile gloves in operating theatres, it is usually to protect myself from infection	Beliefs about consequences	3.98 (1.12; 56)	4.13 (0.97; 137)	0.93 (0.355)
Wearing fewer non-sterile gloves in operating theatres would put me at risk	Beliefs about consequences	2.32 (1.18; 56)	3.61 (1.17; 137)	6.94 (<0.001)*
Reducing unnecessary gloves would have an impact on the environment	Beliefs about consequences	3.96 (1.06; 106)	3.81 (1.03; 221)	−1.24 (0.215)
Reducing unnecessary gloves is likely to happen in my hospital’s OTs	Optimism	3.46 (0.95; 106)	3.21 (1.04; 220)	−2.11 (0.035)*
I plan to use fewer unnecessary gloves during surgery; there is a need to reduce unnecessary gloves in this hospital (Cronbach’s α = 0.835)	Intentions	4.32 (0.85; 56)	3.66 (1.07; 141)	−4.12 (<0.001)*
I believe the OT should become more environmentally friendly	Goals	4.76 (0.61; 106)	4.44 (0.79; 223)	−3.58 (<0.001)*
Thinking about using fewer gloves in operating theatres makes me feel anxious/unsafe (Cronbach’s α = 0.801)	Emotion	1.97 (1.19; 56)	3.01 (1.13; 137)	5.70 (<0.001)*
Overall disgust score (out of 7)	Emotion	4.92 (1.24; 106)	5.29 (1.05; 223)	2.81 (0.005)*
I feel pressured by my colleagues to use gloves when I don’t think it is necessary to do so; not wearing gloves for activities like moving a patient would be criticised (Cronbach’s α = 0.813)	Social influences (injunctive)	2.53 (1.26; 106)	3.11 (1.08; 219)	4.30 (<0.001)*
I think that in some cases my colleagues do not wear gloves when they should have	Social influences (descriptive)	2.54 (1.38; 106)	3.52 (1.19; 223)	6.60 (<0.001)*
I think that in some cases my colleagues wear gloves when they don’t need to	Social influences (descriptive)	4.24 (0.99; 106)	3.79 (1.10; 223)	−3.51 (<0.001)*
I believe my Trust is competent/committed at protecting me/patients from infections (Cronbach’s α = 0.879)	Social influences	3.73 (0.77; 106)	3.66 (0.76; 223)	−0.77 (0.440)

* significant results (under 0.05).

OT, Operating Theatre; TDF, Theoretical Domains Framework.

**Table 4:** Influences on unnecessary glove use (yes-no binary response; N=217)

Influences on non-sterile glove use with binary responses	TDF domain	n responding ‘yes’—of those who use gloves appropriately (N=69) (%)	n responding ‘yes’—of those who use gloves unnecessarily (N=148) (%)
I often put on a pair of gloves in the operating theatre without thinking about it	Memory, attention and decision processes	11 (16%)	80 (54%)
I think carefully about whether a pair of gloves is necessary when I’m in the operating theatre	Memory, attention and decision processes	43 (62%)	105 (71%)
I think about the impact of using gloves on the environment	Behavioural regulation	41 (59%)	79 (53%)
I am in control of how many gloves I use in the operating theatre	Beliefs about capabilities	35 (51%)	94 (64%)
It is easier to wear gloves than sanitise hands in an operating theatre	Environmental context and resources	7 (10%)	60 (41%)
It is easier to sanitise hands than wear gloves in an operating theatre	Environmental context and resources	24 (35%)	59 (40%)
I feel more competent at performing my role when wearing gloves	Social/professional role and identity	8 (12%)	70 (47%)
I wear gloves as it is better to be safe than sorry	Emotion	10 (14%)	86 (58%)
It would be unhygienic not to use gloves when moving a patient who is under general anaesthetic where there is no visible soiling	Emotion	4 (6%)	45 (30%)
It would be unhygienic not to use gloves when putting socks onto a patient	Emotion	12 (17%)	49 (33%)
It would be unhygienic not to use gloves when shaving the area of the operation (in a non-intimate area and not visibly dirty)	Emotion	19 (28%)	63 (43%)
It would be unhygienic not to use gloves when holding a sealed sample pot	Emotion	7 (10%)	20 (14%)
Have you had training on when to use gloves?	Knowledge	25 (36%)	85 (57%)
Are you aware of any guidelines on when to use non-sterile gloves?	Knowledge	31 (45%)	83 (56%)

TDF, Theoretical Domains Framework.

#### Theme 1—sustainability is not prioritised in the organisation

Participants felt the NHS is focused on other priorities, mainly financial and time savings, rather than sustainability initiatives. They discussed the significant stress currently faced by the NHS due to staff shortages, financial constraints and long patient waiting times. This results in a lack of time and resources from the wider organisation to engage with issues that are not as pressing or urgent. Some participants felt that this lack of encouragement from the NHS led to individuals also not prioritising sustainability.

Unfortunately, I think things come down to finances and they come down to an NHS that is failing to deliver… And that pot of money is going to be ever pressured*.* (L, registrar surgeon)

Many participants also discussed a culture of wasteful practices within the NHS as a whole, making it challenging to monitor the usage and wasting of specific items: “nobody in the NHS really keeps tabs of what’s being used” (B, consultant surgeon). This results in individuals engaging in wasteful practices without feeling any direct personal consequences. In the survey only about half of the respondents agreed that they think about the impact of gloves on the environment (55%). Staff have other priorities, both in the context of the NHS (patient turnover times and money) and in their role, including the energy and time required to change established routines or add new responsibilities, although survey respondents did agree overall that the OT should become more environmentally friendly (mean 4.5±0.8).

I think most people…can't be bothered with that because they're so unhappy about what they're doing, how they're being paid, about the whole situation, that if you tell them, ohh you need to save the environment and use less gloves. That’s the least of their problems. (A, senior nurse)

Individuals are also not incentivised or externally motivated to reduce glove use. There are no repercussions for wasting more and no benefits for wasting less. This is despite the fact that, presumably, using fewer gloves and indeed reducing waste overall would also lower NHS costs.

What is the incentive for an individual surgeon or team to reduce waste, and the answer, apart from moral wellbeing, is nothing*.* (B, consultant surgeon)

Awareness of cost of gloves and incineration of waste etc- many ignorant to this. (Nurse in survey)

#### Theme 2—patient outcomes are the absolute priority in OTs

For staff in OTs, patient safety is the top priority, taking precedence over goals related to waste reduction and cost minimisation. A key influence contributing to this prioritisation is fear of potential complications and adverse patient outcomes. Therefore, while acknowledging that using more gloves can be wasteful, most participants emphasised that glove use is an important protocol for protecting patients, specifically when handling anatomical areas with a high risk of infection. In the survey, those who wore gloves unnecessarily agreed significantly more that protecting patients is a key reason for wearing NSGs (p<0.001).

Every single time [staff double glove] because we're dealing with the central nervous system, where if there’s any infection, it can lead to ventriculitis, encephalitis, meningitis, which is extremely life-threatening. (P, registrar surgeon)

This fear of causing harm to patients often translated to resistance to modify established behaviours in OTs. If a system has been working and is something staff are comfortable with, they typically would not want to disrupt these routine practices. Consequently, despite goals and opportunities provided by the NHS to adopt more sustainable practices, staff may be resistant to change:

If something works well for me in the operating theatre and has been working well for my patients in terms of reduced morbidity, and generally good outcomes, I'm a bit reluctant to change. (K, consultant surgeon)

Another factor that may contribute to the increased waste in OTs is the time-sensitivity of many procedures. In time-critical situations, any additional time expended could detrimentally affect patient outcomes. Thus, some participants believed that if engaging in preparatory behaviours such as donning NSGs before they are actually required could even marginally expedite surgical procedures, the benefits would outweigh any possible adverse environmental consequences:

as soon as you enter that theatre, you know you have to be super quick, like a robot. Cause you don't have time to waste cause the patient is bleeding and obviously any delay can make a significant difference to their outcome… if they ask you for something, then you can't just go and get gloves and then put that on and then, you know, give them the instruments. You just have to be standing there ready to give anything they ask you for*.* (M, junior nurse)

#### Theme 3—social dynamics within the OT can play a role in increasing waste

OTs are a complex environment whereby many individuals and factors interplay to ensure efficiency; each team member has a specific role to ensure the smooth execution of surgery. Strong team dynamics, with clear communication, often result in less waste. However, situations where team members are less familiar with each other or lack confidence to provide constructive feedback can result in increased waste.

Despite efforts to reduce hierarchy within OT teams, participants reported that individuals with more authority, such as consultant surgeons and senior nurses, often use their influence to direct others’ actions. This dynamic can make junior team members feel reluctant to express their opinions or to disagree with senior staff, fearing it may impact their relationships. Many participants reported that issues with team dynamics included difficulties in voicing concerns about waste reduction or facing reprimands from authority figures when attempting to minimise waste. This was relevant both within groups (senior compared with junior nurses) and across groups, particularly with nurses.

Nursing staff reprimand you if you don’t have gloves on to move a patient (Surgeon in survey)

Some participants emphasised the importance of overall team atmosphere and camaraderie in reducing waste. When team members are familiar with each other and implicitly understand each other’s work styles, it reduces waste due to streamlined and efficient processes.

with vascular, we have a nurse who’s been working for like 26/27 years. And same with the surgeon. So they know each other really well. So they sort of work together to make sure that they're not wasting anything*.* (M, junior nurse)

In comparison, participants also described teams with a less supportive atmosphere, which resulted in more waste. A negative environment leads to team members aiming to prevent disagreements by mitigating any potential issues that may cause conflict. This sometimes led to more wasteful practices.

Mr X is doing something, we know from experience he will blow his lid if that’s not available on the set. Let’s get this ready in case, everything needs to be ready, everything needs to be perfect. (L, registrar surgeon)

Related to this, lack of clear communication among team members sometimes increases waste. For example, when nurses anticipate the needs of the operating surgeon without the surgeon’s preferences being explicitly stated, they might unnecessarily put on gloves.

the runners might think, OK, I've finished that bit and then the boss is like, actually, no, sorry, I need you to reposition them. Then they put on a new pair of gloves, blah, blah, blah, blah. You might waste many, many pairs in a thing*.* (L, registrar surgeon)

In the survey, those who use gloves unnecessarily were significantly more likely to report feeling pressured by colleagues to use gloves when they did not believe they were needed (p<0.001). All participants agreed that, in some cases, colleagues wear gloves unnecessarily (mean 3.7±1.4); however, this view was expressed more strongly by those who used gloves appropriately (mean 4.2±1.0). These findings suggest that individuals who overuse gloves perceive both a greater expectation for others to wear gloves and a heightened social expectation to wear gloves themselves.

#### Theme 4—lack of availability or poor quality of facilities results in increased waste

Sometimes facilities are lacking in either their availability or quality, which increases waste. NSGs can be poor quality, and tears or other faults result in requiring more gloves, thus increasing waste. Glove packaging also leads to waste: often more gloves are taken than required as the dispenser is unreliable, and once gloves are out of the dispenser they must be used or disposed of for hygiene reasons.

we have the glove dispenser thing, and when we're trying to take a pair of gloves, sometimes one comes out and just falls. And I've noticed people just leave that on the floor and they ignore it or they just throw it away (A, senior nurse)

Furthermore, gloves were described by most participants as being readily available and easy to access within theatres and in key areas where they may be required, while any alternatives (handwashing or predominantly hand sanitiser) are less accessible. Hand sanitiser is often hard to find in OTs or sometimes not present at all. Therefore, participants noted that even if they wanted to use an alternative, often this was not possible. In the survey, more respondents who wore gloves unnecessarily (41%) reported that it is easier to wear gloves than sanitise hands in the OT, compared with only 10% of those who had appropriate glove use.

#### Theme 5—importance of clear guidelines and training in NSG use reduction

Although many aspects of OTs have clear standards and processes, and general guidelines and policies for general glove use exist, there are no clear policies for glove use which are specific to OTs, and participants were not aware of whether they existed or not. For survey respondents, only 53% knew of guidelines for glove use. Some participants felt that their Trust’s policy was to use gloves as a default.

My health trust take to the policy to wear gloves and aprons when transferring patients no matter what! (Surgeon in survey)

Despite expressing willingness to follow such guidelines if available, participants were not motivated to find them, and instead believed it was important to be guided by factors such as ‘common sense’.

And then it’s just about using your common sense, at least to me. And it’s more about common sense than knowing exactly what to wear and when. (A, senior nurse)

Many participants mentioned receiving training on glove use, with some nurses specifically advocating for increased training on when to use and not use gloves, especially to improve infection control practices.

we encourage that it [gloves] shouldn't be necessarily used, unless you really are handling infected something…because we end up contaminating other items, equipment, and other people who hold it with their hands and get reinfected. (N, senior nurse)

However, in the survey, only 51% had received training on glove use, and some participants said that training was not adequate. Some participants also mentioned that initial training in medical schools reinforced glove use by requiring their use in their training for patient interactions.

No one is reminded or trained properly in it and so default to wearing them. (Surgeon in survey)

Stopping medical schools from enforcing students to wear gloves when simulating clinical examinations*.* (Surgeon in survey)

Furthermore, some training received by participants seemed contradictory. One participant was instructed to wear gloves whenever touching a patient, but was then told not to wear gloves when checking the patient’s pulse.

when I used to go to uni [you were taught] that whenever you come in contact with patients, you wear gloves…Unless obviously you have to feel their pulse cause then you can't wear gloves*.* (M, junior nurse)

#### Theme 6—individuals’ preferences and habits

Given the lack of well-communicated and clear guidelines for glove use in OTs, decisions about glove use tend to be made by individuals. Some participants openly acknowledged that in certain situations, whether to wear gloves or not was decided purely by personal preference.

Many participants mentioned the habitual nature of putting on gloves, influenced either by observing others in OTs or their own experiences. In the survey, 54% of those who use gloves unnecessarily felt that they often put on gloves in the OT without thinking about it, compared with 16% of those who use gloves appropriately. Typically, participants did not consciously think about their glove use, and it was only through discussing different situations where glove use might not be necessary that they realised how often gloves were used, both by themselves and the whole team. This habitual glove use was often triggered by certain situations, many routinely occurring during surgeries. For example, simply entering the OT would prompt glove use, as one participant described:

they will put gloves on almost immediately, they're not doing anything or touching anything.… I see it a lot with some of the medical students they'll walk in [to the operating theatre], they’ll put gloves on and see, you don't need them cause you’re not doing anything*.* (R, consultant anaesthetist)

Other participants felt that they were conscious of whether to wear gloves in each situation. In the survey, 68% of participants agreed that they thought carefully about the necessity of each pair of gloves in the OT, with 71% of those who use gloves unnecessarily agreeing with this. In the interviews, it was those who identified as caring about sustainability who were more inclined to consider reducing their own waste and, therefore, described being more conscious and thoughtful about their use of gloves and other disposables. In comparison, participants who did not identify with this mindset mentioned that they had not previously considered their glove use prior to the interview. Thus, fostering a sustainability-focused identity could serve as a facilitator for reducing glove use.

I think I judge every single situation everyday because it’s a huge waste…I worry about the environment, all day everyday. So that’s a priority for me*.* (A, senior nurse)

I do use a lot of gloves, to be honest…I have a habit as well of using gloves all the time…personally I haven't actually thought about, you know, gloves and wasting*.* (M, junior nurse)

However, some participants, even when it was a conscious decision whether to wear gloves, often would decide to wear the gloves unnecessarily due to fear of infection for themselves rather than for the patient. In the survey, participants agreed that their use of NSGs was primarily to protect themselves from infection (mean 4.1 ± 1.0).

Everyone has a preference in using non sterile gloves especially working in the theatres, mostly it is to protect themselves*.* (Nurse in survey)

Survey respondents who use gloves unnecessarily also indicated that they experienced greater anxiety about reducing glove use than those who wore them appropriately (p<0.001). This fear was exacerbated by the COVID-19 pandemic, when gloves and other personal protective equipment were widely used, especially in hospitals. Survey respondents agreed that the pandemic had indeed increased glove use (mean 4.2±1.2).

I remember before people used to wear 3, 4, 5 gloves, people used to say, I'm getting protected…we've become paranoid…definitely people are wearing gloves more now, covid has changed our mentality. (N, senior nurse)

You have to remember what we went through with covid and with the amount of bodily fluid we had to deal with… A lot of us are still dealing with mental or physical health issues from that period in time. (Nurse in survey)

This manifested itself in wanting to be ‘better safe than sorry’: wearing gloves was considered the more hygienic and less risky option (for personal protection) and therefore, it was better to just wear gloves. This was not always a decision that participants described consciously making in every situation. However, when asked why they wore gloves, some participants would describe a feeling of comfort and safety from wearing them. In the survey, 58% of those who wear gloves unnecessarily agreed that they wear gloves as ‘it is better to be safe than sorry’ compared with only 14% of those who wear gloves appropriately.

I think I use them a bit more than what I should, just so I can feel better about. If I'm being very honest…Just so I can maybe feel a bit more comfortable and safer, I guess, when I'm working (D, SHO surgeon)

The use of non sterile gloves gives us a sense of security and peace of mind in clinical environment knowing we are protecting ourselves and our family that we are not take [sic] home possible bacteria/germs/microorganisms etc. (Nurse in survey)

Some individual differences appeared to align with experience levels. Junior staff tended to overuse gloves much more than their more experienced counterparts, often due to fear of infection, a desire to act appropriately (and believing that wearing gloves is the appropriate action), or a lack of confidence in when to use and not use gloves. In contrast, those with longer experience or higher seniority tended to feel more comfortable and were less worried about doing things ‘correctly’ or strictly following infection control practices. This was reflected as a general trend in survey respondents, where 47% of those who wear gloves unnecessarily agreed to feeling more confidence in their roles when wearing gloves, compared with 12% of those who wear gloves appropriately. Furthermore, participants with under 5 years of experience reported higher unnecessary glove use than those with over 5 years of experience (means 3.5±1.9 vs 2.9±1.4; t=3.6; p<0.001)

I’m probably part of the slightly older generation who examine without gloves and then just wash my hands*.* (C, registrar surgeon)

#### Theme 7—suggestions for interventions

The main suggestions for interventions to reduce glove use identified in the survey comments were: appointing a champion for glove use reduction, placing physical prompts to remind staff of appropriate glove use, providing more education and training on the impacts of glove use, clarifying when gloves are necessary, and increasing access to alternatives such as alcohol gel.

Have the infection control nurses doing road shows on all the wards & theatres & icu that it isn’t necessary*.* (Surgeon in survey)

Put a counter in the glove dispenser.” (Surgeon in survey) “We need more alcohol gel in theatre to clean hands after touching surfaces or patient to encourage less glove use*.* (Anaesthetist in survey)

Some participants also suggested interventions based on other similar initiatives that they had heard about or experienced in their own hospital. A few respondents also mentioned that they had independently tried to reduce their own glove use, which may not always have been done in hygienic ways.

My hospital is working on reducing gloves and its a massive paradigm shift. Really hard. (Nurse in survey)

Currently Gloves Off campaign in our trust*.* (Anaesthetist in survey)

I try and use one set for as long as possible by sterilise with alcohol gel if there is no soiling. Change with visible or possible soiling. (Anaesthetist in survey)

Many survey respondents also highlighted other sustainability issues needing attention, specifically: reducing waste, unnecessary equipment and packaging; increasing recycling efforts; managing energy use; and increasing the use of reusable equipment and fabrics (gowns, drapes).

How the equipment is wrapped, how the rubbish is managed etc, the plastic equipment packaging - none of us know which elements even are recyclable, some are paper and plastic and nobody will stand there thinking which goes where and which is recyclable (if not marked) because we have more important things to do and we're constantly under other pressures. (Nurse in survey)

## Discussion

Overall, 67% of surveyed participants reported using gloves unnecessarily in OTs. This study identified key influences for this behaviour, including a lack of incentives from the NHS to reduce glove use, focus on patient safety, lack of standardised training or clear guidelines for appropriate glove use in OTs, social influences (particularly having an unsupportive team environment), unavailability of alternatives to gloves and individual influences such as preferences. Additionally, many staff members reported feeling more protected and safer when using gloves, leading to glove-wearing becoming a habitual practice for many.

This study identified a culture of waste within the NHS, and a lack of consideration of sustainability. Interviewees described the UK NHS as struggling with significant issues, such as staff shortages, resulting in a lack of time or resources dedicated to less pressing concerns such as reducing waste in OTs. Similarly, previous research has found that when there are personnel shortages and high workloads, sustainability in OTs becomes a lower priority,^22^ and that lack of time and a busy environment are factors in less optimal personal protective equipment use, including gloves, in emergency departments.^24^ These contextual factors result in individuals having lower cognitive resources, associated with less environmentally friendly behaviour and also reduced hand hygiene compliance.^36 37^ Therefore, individuals have little motivation for reducing their glove use, or time to dedicate to solutions for this issue. Future interventions should ensure to address this context, for example through involving wider management in encouraging reduction of glove use, and ensuring that cognitive burden is reduced with clear examples of when to use and not use gloves.

Social influences were found to be an important contributing factor to unnecessary glove use in both the interviews and survey, confirming prior findings of the literature related to the impact of social influences on pro-environmental behaviour.^38^ Participants wearing gloves unnecessarily were more likely to report feeling pressured by colleagues to do so and expressed concern about being criticised if they did not wear gloves in certain situations. In the interviews, participants described that being reprimanded by colleagues resulted in a preference to wear gloves to avoid conflict. Previous studies of glove use in other contexts have also found that healthcare workers follow the practices of colleagues, and that this results in the culture being to routinely use gloves, regardless of the specific situation.^24 25 39^ A recent intervention to reduce glove use on hospital wards in Australia changed the social culture around glove use using a multi-component design, for example using badges with the campaign logo worn by key staff, reminders during daily nurse meetings, glove shaped baked goods and even a related music video recorded by staff, resulting in a highly successful reduction in unnecessary glove use from 60% to 23%.^40^

A further reason why unnecessary glove use may be so common is because gloves are more accessible than alternatives, as found in both the interviews and survey. Interviewees reported that hand sanitisers are not as prominently displayed as glove boxes in OTs, and may not be present at all. This has also been found in observational studies of sustainability in OTs.^41^ To counteract this barrier and make hand sanitisation just as convenient, interventions should ensure that alternatives to gloves are equally, if not more, available and visible. Additionally, prompts encouraging the use of hand sanitiser over gloves at the point of use could help reduce habitual behaviour being triggered. Many survey respondents suggested visual prompts and increased visibility of sanitisers as potential interventions to reduce unnecessary glove use, and indeed these have been utilised in other interventions to reduce glove use or increase hand hygiene successfully.^40 42 43^

Another key influence on glove use for which prompts on glove boxes may be useful was how habitually they are used, as we found that habits were a key influence on glove overuse. In the survey, 54% of those who use gloves unnecessarily felt that they often put on gloves in the OT without thinking about it, compared with just 16% of those who use gloves appropriately, and in interviews participants discussed that use of gloves was not a conscious decision. Glove use was often prompted by routine tasks, even simply walking into the OT. Habits have been reported as a key reason for glove use in other healthcare contexts in previous studies.^12 24 25^ We found in interviews that those with values of caring about the waste they generate and its environmental impact also mentioned being more conscientious about their glove use in every situation, confirming prior literature that environmental self-identity is closely associated with sustainable behaviour.^44^ Other research has found that conscious consideration of one’s waste in the OT can lead to deliberate efforts to reduce it, thereby minimising waste during surgeries.^27^ Thus, fostering a sustainability-focused identity could also serve as a facilitator for reducing glove use.

Gloves may also be used habitually due to a lack of knowledge as to when gloves are necessary, and therefore staff may default to using gloves in every situation. In our survey, only 53% of participants knew of guidelines for glove use, and 51% responded that they had received training on glove use. Previous research of glove use in other healthcare contexts also has found that even when staff say that they are aware of policies or guidelines around glove use, they may use them unnecessarily in practice, suggesting that awareness of such guidelines does not result in day-to-day application of them.^45^ Despite this, most hand hygiene and glove use interventions focus on education, which may not take habits into account.^46^,^48^ One previous intervention (in the emergency department and radiology) compared intensive education in one group to only stickers on glove boxes in another, and found that while education plus stickers resulted in a 63% reduction in unnecessary glove use, stickers alone resulted in a 54% reduction, suggesting that prompting habit change is a key factor in reducing overuse of gloves.^42^

A further reason for the tendency to wear gloves unnecessarily is a belief that it is ‘better to be safe than sorry’, a belief which arose from the interviews and was associated with more unnecessary glove use in the survey. This sentiment may be driven by emotions associated with glove use, such as feeling at risk without them: survey respondents who used gloves unnecessarily agreed more that they would feel anxious or unsafe without gloves, and that wearing fewer gloves would put themselves at risk. These emotions are also found in literature of reducing glove use in other contexts. For example, an interview study found that glove use was driven by emotions such as feeling safer, more confident and fear of infection.^12^ They also found that disgust was a key factor, however we did not find that disgust or concerns about hygiene were relevant, showing that there may be differences between glove use in OTs compared with other healthcare contexts. It is interesting that feeling more comfortable and safer were described as reasons to use gloves, as in fact glove use is linked to more cases of cross-contamination.^15^,^18^ This is suggested in previous research which has found that healthcare staff view gloves as a complete barrier to infection and that they feel less clean after using alcohol gel despite gel being more effective for hand hygiene.^39^ Providing training from credible sources, such as infection control departments, should help alleviate these concerns, and this training could be corroborated by a ‘sustainability champion’ for advising on appropriate glove use at helpful moments, which has been implemented in other similar interventions.^47 49^

Several limitations of this research should be acknowledged. Participants were more likely to be those with an interest in environmental sustainability, potentially making them more motivated to engage in behavioural change than the broader population. However, 8% of survey respondents were ambivalent towards or disagreed with the statement that the OT needs to become more environmentally friendly, suggesting the sample was not exclusively composed of highly motivated advocates. As participation was voluntary, self-selection bias cannot be fully eliminated, and findings should therefore be interpreted as descriptive and hypothesis-generating rather than population-representative. Recruitment via multiple channels (Royal Colleges, professional newsletters, hospital posters and clinical networks) and the inclusion of a monetary incentive were intended to maximise reach and reduce dependence on any single recruitment pathway. A conventional response rate could not be calculated, as the open multi-channel recruitment strategy makes it impossible to determine the total number of eligible individuals exposed to the survey; this is methodologically expected in this type of design rather than a flaw (Zimba & Gasparyan, 2023). Generalisability is further limited by geographic concentration of participants, with all interviewees and the majority of survey respondents based in England, predominantly in London. These limitations are partially mitigated by the consistency of findings across the qualitative and quantitative phases, which increases confidence that the patterns identified reflect genuine behavioural phenomena rather than sampling artefacts.^35^ A key strength of this research is the mixed methods design and the use of the TDF across both parts of the research, which is designed to capture all relevant behavioural determinants and is linked into a wider system of the Behaviour Change Wheel, which can guide the translation of these findings to practical and effective interventions.

## Conclusions

Overall, 67% of participants reported using gloves unnecessarily in OTs. Across two methodological approaches, key barriers for reducing this unnecessary use included habitual behaviour, organisational culture, social influences and unclear guidelines. These learnings should guide the development of relevant and effective interventions, such as clearly communicating when to use and not to use gloves, changing the social culture to prioritise waste reduction, supporting habit change, increasing accessibility to hand gel and appointing sustainability champions to facilitate change.

## Supplementary material

10.1136/bmjopen-2025-102835 online supplemental file 1

## Data Availability

Data are available upon reasonable request.
