# Supplementary material for: Exploring the overuse of non-sterile gloves in operating theatres: a cross-sectional survey and interview study
Source: BMJ Open. 2026 Jun 2;16(6):e102835. doi: 10.1136/bmjopen-2025-102835 (PMC13239380; doi:10.1136/bmjopen-2025-102835)
Supplement: online supplemental file 1 [file bmjopen-16-6-s001.docx]

# Exploring the overuse of non-sterile gloves in operating theatres: A cross-sectional survey and interview study

Carys Batcup^1,2^, Aws Almukhtar^3,4^, Aarya Menon^4^, Daniel Leff^3^, Gaby Judah^4^, Pelin Demirel^1^, Talya Porat^1^

^1^Dyson School of Design Engineering, Imperial College London, Imperial College Road, South Kensington, London, SW7 2DB

^2^Faculty of Social and Behavioural Sciences, University of Amsterdam, Nieuwe Achtergracht 129, Amsterdam, 1018 WT

^3^Department of General Surgery, Imperial College Healthcare NHS Trust, St Mary’s Hospital, London

^4^Department of Surgery and Cancer, Imperial College Healthcare NHS Trust, St Mary’s Hospital, 10th Floor Queen Elizabeth Queen Mother Building, W2 1NY, London

Correspondence

Carys Batcup

Faculty of Social and Behavioural Sciences, University of Amsterdam

Nieuwe Achtergracht 129, Amsterdam, 1018 WT

[c.a.batcup@uva.nl](mailto:c.a.batcup@uva.nl)

## Supplementary files – index

**Supplementary methods**

Interview schedule page 2

All survey questions page 5

**Supplementary results**

Survey demographics in full page 7

### Supplementary file 1 - Interview schedule


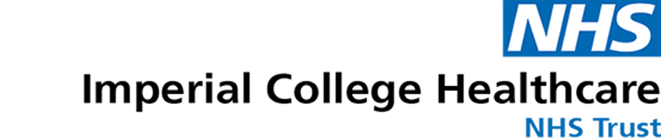


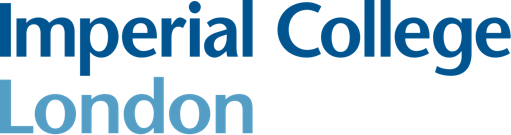


**Topic Guide**

***A qualitative study to engage key stakeholders to identify the facilitators and barriers to the adoption of green surgery practices and co-design interventions***

**Introduction**

Thank you for taking the time to talk with me today.

The aim of this interview is to understand more about barriers and facilitators to reducing the environmental impact of operating theatres in the UK.

Please feel free to skip over any questions you do not wish to answer, and to answer in as much or as little detail as you would like. Your responses will remain anonymous. Let me know if you would like to take a break or stop the interview at any point.

Do you have any questions?

I’ll start recording now, is that okay?

*Start recording and transcribing*

*Ask everyone opening questions in part 1, in part 2 ask the appropriate questions for that role, and ask everyone part 3 closing questions*

**PART 1: OPENING QUESTIONS.**

**Can you start by telling me a little bit about yourself. What is your current professional role?**

**Which operations do you perform or participate in?**

E.g. which specialties, which types of operations, duration etc

**PART 2a: GLOVES.**

**Thank you. The next questions are about use of gloves in operating theatres.**

| **Question** | **TDF** |
| --- | --- |
| **Can you talk me through your use of gloves in an operating theatre? So starting with preparing the room for the patient, or setting up the room for the patient.** | Knowledge |
| **When do you first put on gloves?** |  |
| How do you choose which type of glove to use, based on which circumstance? E.g. double gloves vs single gloves, sterile gloves |  |
| What are surgeries which use more gloves vs not as many gloves? Are there differences? |  |
| How long do you wear a pair for? |  |
| What are the reasons for removing a pair or putting on a new pair? – e.g. when contaminated, different stages of the operation, bad quality gloves that rip |  |
| **When do you think it is necessary to use gloves?** |  |
| When is it not necessary? E.g. when moving a patient pre-surgery |  |
| What are the differences between times you’d use a pair of gloves vs washing your hands or using alcohol hand rub? |  |
| Are there any times when using gloves might be seen as unnecessary, but you still choose to use them? Why? |  |
| **Do you think there is a need to reduce the number of gloves used in your team or hospital? - just in the operating theatre** | Intentions |
| Do you think efforts should be made to reduce this? Can you elaborate? |  |
| **Had you thought about your use of gloves before?** |  |
| To what extent is this something you plan to work on? Have you thought about the environmental impact of gloves? In what way? |  |
| **Do you think the use of gloves changed compared to before Covid?**  In what way? Why? Do you / others use more gloves? | Other (Covid) |
| **If the number of gloves was reduced, what impact do you think this would have on the environment?** | Beliefs about consequences |
| **And on healthcare? Patients? Quality of care given to patients?** |  |
| **Can you describe any training or teaching you have had on reducing glove use, or about when it is necessary to use gloves?** | Skills |
| **Do you know of clear guidelines for reducing the use of gloves in surgery?** | Knowledge |
| If yes - What do these guidelines recommend? |  |
| Where do you find/get this knowledge? (is it from hospital/trust/elsewhere?) How readily available is this information? Are there any challenges to accessing this information? |  |
| Do many people know about this? |  |
| **The guidelines from the RCS state that ‘gloves are only necessary for contact with bodily fluid, non-intact skin, and mucus membranes’** | Knowledge |
| **Did you know about this guideline?** |  |
| How do you feel about this? Do you think this is appropriate or not?  Refer back to what they said earlier about when you should use gloves (if it differs) |  |
| **Whose role or responsibility is it to decide when to use gloves in the operating theatre?** | Social/ professional role and identity |
| Is it the responsibility of each individual, or someone specific? Profession? Grade? Why them/ you? |  |
| Whose role or responsibility do you think it should be? |  |
| Does it depend on: double vs single gloves? Sterile vs non-sterile gloves? |  |
| **Do you think the hospital/management support efforts to reduce the number of gloves?** | Social/ professional role and identity |
| In what way? Or - Why not? How could it be better? |  |
| Is there a difference between different points in an operation/ double gloving vs single |  |
| **Would reducing single-use items fit into your workplace culture?** | Social influences |
| Would your team members in the operating theatre support reducing the amount of gloves? |  |
| Does this differ based on the point in the operation or type of operation? (give examples from earlier) |  |
| Are there team members who would disapprove of not wearing gloves in situations where they may not be necessary? Why? Does this differ in any different hospitals you’ve worked in? |  |
| Do you feel this influence from colleagues affects your choice as to whether to *double* glove or not? |  |
| **We want to know whether any aspects of the clinical area make it harder to use fewer gloves. For example, when going to move a patient or moving instruments, Are gloves more readily available than handwashing/hand sanitiser?** | Environmental context and resources |
| Where is the hand sanitiser kept? Where are gloves kept? Would you use what is closer, if both are possible to be used in a particular circumstance? |  |
| **Do you feel capable of reducing your use of gloves?** | Beliefs about capabilities |
| If not, what makes it harder for you to do this? |  |
| **Do you ever discuss or reflect on the use of potentially unnecessary gloves in the surgeries you have done?** | Behavioural regulation |
| **If you think about using fewer gloves in surgeries, how does that feel?** | Emotion |
| E.g. anxious, motivated. Does this depend on which instances you'd be reducing your use of gloves? |  |
| **Do you ever use these items out of habit, despite intending to use them less?** (only if they described an intention to use them less) | Memory, attention, decision |
| **Do you know of any incentives for reducing gloves in operating theatres?** | Reinforcement |
| **Do you think it is likely that the use of gloves can be reduced in your hospital?** | Optimism |
| Why/why not? |  |
| **Do you think it is likely that you will reduce your use of gloves in surgery?** | Optimism |
| Why/why not? |  |

**PART 3: CLOSING QUESTIONS.**

**Do you anticipate any further barriers to trying to change these behaviours in the operating theatre?**

**Are there any other factors that help you to use fewer gloves?**

Something that already exists e.g. a poster or other campaign, or a particular colleague, or your behaviour/values outside of work, RCS checklist

Do other campaigns e.g. hand hygiene within the Trust or a reduction in desflurane (pick suitable example) influence your behaviour in the operating theatre/in this area?

**Do you have any ideas about how the implementation of measures to reduce the environmental impact of operating theatres can be improved? Is there one thing that would make it easier or motivate you and others to do so?**

Some ideas are: Support or feedback from colleagues; education around impact; incentives

**And finally, this has been about the operating theatre. How might this relate to reducing your impact in other parts of your clinical role?**

E.g. for nurses - Are there different things that make it easier or harder to reduce unnecessary glove use outside of theatre compared to in theatre?

**This is one stage of the project, but we are hoping to look at other areas of the operating theatre in future stages - do you have any other areas you think are important?**

## Supplementary file 2 - All survey questions

| **TDF/ other domain** | **Survey item** | **Response options** | **Nurses** | **Surgeons** | **Anaesthetists** |
| --- | --- | --- | --- | --- | --- |
| Behaviour | I use non-sterile gloves in operating theatres | 1 (never) to 5 (always) | X |  | X |
|  | In the last month, I used non-sterile gloves in the operating theatre when…  touching the patient |  | X | X | X |
|  | I come into contact with bodily fluids, mucus membranes or non-intact skin |  | X | X | X |
|  | I am unsure if there is risk of infecting myself |  | X | X | X |
|  | touching visibly contaminated equipment |  | X | X | X |
|  | touching clean sealed specimen pots |  | X | X |  |
|  | using the whiteboard |  | X |  |  |
|  | moving a patient when there is no visible soiling |  | X | X | X |
|  | opening a door |  | X |  |  |
|  | using the computer |  | X |  |  |
|  | removing a blood pressure cuff |  | X |  |  |
|  | touching an IV bag |  | X |  |  |
|  | moving a drip stand |  | X |  |  |
| Beliefs about behaviour | I only use gloves when it is necessary to do so | 1 (disagree) to 5 (agree) | X | X | X |
| Memory attention and decision processes | I often put on a pair of gloves in the operating theatre without thinking about it | Tick to indicate agreement, or leave blank to indicate disagreement | X | X |  |
| Memory attention and decision processes | I think carefully about whether a pair of gloves is necessary when I'm in the operating theatre |  | X | X |  |
| Behavioural regulation | I think about the impact of using gloves on the environment |  | X | X |  |
| Beliefs about capabilities | I am in control of how many gloves I use in the operating theatre |  | X | X |  |
| Environmental context and resources | It is easier to wear gloves than sanitise hands in an operating theatre |  | X | X |  |
|  | It is easier to sanitise hands than wear gloves in an operating theatre |  | X | X |  |
| Social/ professional role and identity | I feel more competent at performing my role when wearing gloves |  | X | X |  |
| Emotion | I wear gloves as it is better to be safe than sorry |  | X | X |  |
| Social/ professional role and identity | I want to set an example of when gloves are not needed before/after the surgical procedure |  |  | X |  |
| Knowledge | I am not sure how non-sterile gloves are used in the operating theatre |  |  | X |  |
| Beliefs about consequences | When I wear non-sterile gloves in operating theatres, it is usually to protect the patient | 1 (disagree) to 5 (agree) | X | X |  |
|  | Wearing fewer non-sterile gloves in operating theatres would increase the risk of infection for the patient |  |  |  |  |
|  | When I wear non-sterile gloves in operating theatres, it is usually to protect myself from infection |  |  |  |  |
|  | Wearing fewer non-sterile gloves in operating theatres would put me at risk |  |  |  |  |
|  | To what extent would reducing use of unnecessary gloves in operating theatres reduce the impact of operating theatres on the environment? | 1 (minimal impact on the environment) to 5 (large impact on the environment) | X | X | X |
| Goals | There is a need to reduce unnecessary gloves in this hospital | 1 (disagree) to 5 (agree) | X | X |  |
|  | I plan to use fewer unnecessary gloves during surgery |  |  |  |  |
| Social influences | I feel pressured by my colleagues to use gloves when I don’t think it is necessary to do so | 1 (disagree) to 5 (agree) | X | X | X |
|  | Not wearing gloves for activities like moving a patient would be criticised |  |  |  |  |
|  | I think that in some cases my colleagues do not wear gloves when they should have |  |  |  |  |
|  | I think that in some cases my colleagues wear gloves when they don’t need to |  |  |  |  |
| Knowledge | Have you had training on when to use gloves? | Yes, no, or not sure | X | X |  |
|  | Are you aware of any guidelines on when to use non-sterile gloves? | Yes, no, or not sure | X | X |  |
| Emotion | It would be unhygienic to not use gloves when…  moving a patient who is under general anaesthetic where there is no visible soiling | Tick to indicate agreement, or leave blank to indicate disagreement | X | X |  |
|  | putting socks onto a patient |  |  |  |  |
|  | shaving the area of the operation (in a non-intimate area, and not visibly dirty) |  |  |  |  |
|  | holding a sealed sample pot |  |  |  |  |
|  | Thinking about using fewer gloves in operating theatres makes me feel… | 1 (disagree) to 5 (agree) | X | X |  |
|  | anxious |  |  |  |  |
|  | unsafe |  |  |  |  |
|  | People may feel disgusted in different situations. Below is a list of a few situations, please indicate how disgusted that situation makes you feel:  Seeing some snotty tissues left on the table | 1 (no disgust) to 7 (extreme disgust) | X | X | X |
|  | You see someone sneeze phlegm onto their hands |  |  |  |  |
|  | Watching a person pick their nose |  |  |  |  |
|  | On the underground, you are forced to stand close to someone with body odour and greasy hair |  |  |  |  |
|  |  |  |  |  |  |
|  | Feeling something sticky on a door handle |  |  |  |  |
| Optimism | How likely do you think it is that reducing use of unnecessary gloves in operating theatres could happen in your specialty, in your hospital? | 1 (not likely at all) to 5 (extremely likely) | X | X | X |
| Social influences | How competent do you believe your NHS Trust is in… protecting you from infections? | 1 (not at all competent) to 5 (very competent) | X | X | X |
|  | protecting patients from infections? |  |  |  |  |
|  | How committed do you believe your NHS Trust is to… protecting you from infections? | 1 (not at all committed) to 5 (very committed) | X | X | X |
|  | protecting patients from infections? |  |  |  |  |
| Goals | I believe the operating theatre needs to become more environmentally friendly | 1 (disagree) to 5 (agree) | X | X | X |
| / | Do you have any other comments on reducing non-sterile glove use, e.g. whether this is possible or how it may be encouraged? | - | X | X | X |

### Supplementary file 3 - Survey demographics in full

| **Variable** | **N (%)** |
| --- | --- |
| **Participant type** |  |
| A nurse or operating theatre practitioner (including assistants) | 103 (31%) |
| An operating department practitioner (ODP) or Anaesthesia Associate | 46 (14%) |
| A surgeon | 68 (21%) |
| An anaesthetist | 112 (35%) |
| **Surgeon level** |  |
| Foundation year 1 | 2 (3%) |
| Senior House Officer (F2, CT1, CT2, or equivalent) | 14 (21%) |
| Clinical fellow | 13 (19%) |
| Registrar | 14 (21%) |
| Consultant | 22 (32%) |
| Other | 3 (4%) |
| **Nurse level** |  |
| Theatre manager nurse | 36 (35%) |
| Scrub nurse | 37 (36%) |
| Theatre coordinator | 11 (11%) |
| Theatre nurse or runner | 15 (15%) |
| Anaesthetic nurse | 16 (16%) |
| Other | 10 (10%) |
| **Anaesthetist level** |  |
| Anaesthetist - core training or equivalent | 18 (16%) |
| Anaesthetist - specialist trainee or equivalent | 29 (26%) |
| SAS supervised practitioner and specialty doctors | 1 (1%) |
| Consultant anaesthetist or SAS autonomous | 64 (57%) |
| Other | 0 |
| **Age group, years** |  |
| 18-29 | 32 (10%) |
| 30-39 | 147 (45%) |
| 40-49 | 88 (27%) |
| 50-59 | 47 (14%) |
| 60-69 | 14 (4%) |
| 70-79 | 0 |
| 80+ | 0 |
| **Gender** |  |
| Female | 177 (54%) |
| Male | 149 (45%) |
| Prefer to describe myself as | 1 (0.3%) |
| Prefer not to say | 2 (1%) |
| **Ethnicity** |  |
| Asian/Asian British | 50 (15%) |
| Black/ African/Caribbean/Black British | 13 (4%) |
| Mixed/Multiple ethnic background | 10 (3%) |
| White | 237 (72%) |
| Any other ethnic group, please describe | 14 (4%) |
| Prefer not to say | 5 (2%) |
| **Hospital type** |  |
| Tertiary hospital | 223 (68%) |
| District General Hospital (DGH) | 96 (29%) |
| Rehabilitation, community or 'cottage' hospital | 3 (1%) |
| Private hospital | 16 (5%) |
| Other | 10 (3%) |
| **Specialty** |  |
| Bariatric surgery | 29 (9%) |
| Breast surgery | 69 (21%) |
| Cardiothoracic surgery | 28 (9%) |
| Colorectal surgery | 72 (22%) |
| Emergency surgery | 127 (39%) |
| Endocrine surgery | 19 (6%) |
| Gastric surgery | 35 (11%) |
| General surgery | 129 (39%) |
| Hepato-Pancreato-Biliary surgery | 32 (10%) |
| Neurosurgery | 36 (11%) |
| Obstetrics /Gynaecology/ Obstetrics and gynaecology | 88 (27%) |
| Ophthalmic surgery | 39 (12%) |
| Oral maxillofacial surgery | 50 (15%) |
| Otorhinolaryngology (ENT) surgery | 56 (17%) |
| Paediatric surgery | 67 (20%) |
| Plastic surgery | 68 (21%) |
| Sarcoma and soft tissue surgery | 13 (4%) |
| Trauma and orthopaedic surgery | 107 (33%) |
| Urology | 83 (25%) |
| Vascular surgery | 46 (14%) |
| **Experience in operating theatres** |  |
| Less than a year | 10 (3%) |
| 1 to 5 years | 107 (33%) |
| 6 to 10 years | 69 (21%) |
| 11 to 20 years | 68 (21%) |
| Over 20 years | 75 (23%) |
| **Country** |  |
| Northern Ireland | 1 (0.3%) |
| Wales | 15 (5%) |
| Scotland | 15 (5%) |
| England | 298 (91%) |
| **Where in England** |  |
| North East, including north Cumbria | 7 (2%) |
| North West | 10 (3%) |
| Yorkshire and the Humber | 6 (2%) |
| East Midlands | 4 (1.3%) |
| West Midlands | 30 (10%) |
| East of England | 13 (4%) |
| London | 187 (63%) |
| Kent, Surrey and Sussex | 9 (3%) |
| South West, Peninsula region | 8 (3%) |
| South West: Severn region | 4 (1%) |
| Thames Valley | 14 (5%) |
| Wessex | 6 (2%) |
| **Trained in the UK** |  |
| Yes | 268 (82%) |
| No | 61 (19%) |
| **How long worked in UK** |  |
| Less than a year | 3 (5%) |
| 1 to 5 years | 18 (30%) |
| 6 to 10 years | 8 (13%) |
| 11 to 15 years | 2 (3%) |
| 16 to 20 years | 5 (8%) |
| Over 20 years | 25 (41%) |
