# Supplementary material for: Exploring the overuse of non-sterile gloves in operating theatres: a cross-sectional survey and interview study
Source: BMJ Open. 2026 Jun 2;16(6):e102835. doi: 10.1136/bmjopen-2025-102835 (PMC13239380; doi:10.1136/bmjopen-2025-102835)
Supplement: Supplementary file 2 [file bmjopen-2025-102835.reviewer_comments.pdf]

PEER REVIEW HISTORY

BMJ Open publishes all reviews undertaken for accepted manuscripts. Reviewers are asked to complete a checklist review form and are provided with free text boxes to elaborate on their assessment. These free text comments are reproduced below.

ARTICLE DETAILS

Title (Provisional)

Exploring the overuse of non-sterile gloves in operating theatres: A cross-sectional survey and interview study

Authors

Batcup, Carys; Almukhtar, Aws; Menon, Aarya; Leff, Daniel; Judah, G; Demirel, Pelin; Porat, Talya

---

VERSION 1 - REVIEW

**Reviewer**	**1**
**Name**	**Merry, Alan**
**Affiliation**	**University of Auckland, Dept of Anaesthesiology**
**Date**	**01-Jul-2025**
**COI**	**I am a voluntary helper for the Planetary Accounting Network that promotes environmental sustainability.**

Thank you for the opportunity to review this interesting paper that deals with a very important topic – broadly, the contribution of healthcare to the crisis in sustainability currently faced by humankind.

I should explain that I am not a qualitative researcher, although I have participated in a fair amount of quantitative and mixed methods research. My comments on the qualitative aspects of this work should take that into account and I suggest that a review by a qualitative researcher is essential. I am an anaesthetist, and primarily a quantitative researcher.

I hope my comments will help you to improve this paper – they are offered for your consideration with that objective in mind.

Introduction

The introduction is readable, relevant, well referenced and largely to the point. I wonder whether you should include at least one sentence about the parlous state of the planet and the massive risks we currently face. Given that your paper is about gloves, which are presumably rubber or plastic, the threat they pose is presumably primarily to the Novel Entities Planetary Boundary. I think some reference to the Planetary Boundaries

https://www.stockholmresilience.org/research/planetary-boundaries.html would be appropriate and perhaps a little clarification of the contribution of gloves (rather than waste more generally) on eCO2 and hence the Climate Change boundary as well as Novel Entities (notably including plastics in the oceans, and microplastics in food). This is, after all, why your study matters.

I think another point that needs to be brought out a bit more in your paper, starting with the introduction, and again in the discussion, is the primary purpose of non-sterile gloves (NSGs). You do refer to this, including in your data, but I think it needs to be given more emphasis. As I understand it, these gloves (in contrast to sterile gloves) serve primarily to protect the wearer rather than patients. It is hard to see how non-sterile gloves can inhibit passing bacteria from one patient to another unless they are changed between patients, and indeed between activities. For protecting oneself, the fact that they may be less hygienic than alternatives such as alcohol gel isn't really the only point (although it is also relevant). Is alcohol gel as protective to the clinician as NSGs? I believe that there is also some evidence worth mentioning that NSGs are often contaminated in the open boxes from which clinicians select them (my apologies - I can't put my hand on the references at present). Thus, their use could actually pose a risk to patients.

I wonder whether the discussion of behavioural frameworks and qualitative approaches could be more concise in the Introduction and perhaps expanded upon in the Discussion?

Your aims set the scene for two studies that are inter-related – the interviews informed the survey. This does create a rather lengthy paper with a lot of data to digest. I did find myself at least wondering whether this work should be presented as two studies, one following the other.

Phase 1 - Interviews

I found this section relatively convincing and have little to say other than that the results are very interesting. You appear to have included a reasonable number of patients for interview research, with a good representation from different craft groups. How did you decide on the number 19?

Your comment that you did not involve patients or the public because "patients are not as involved in decision making" was interesting. I suspect patients might have a view on how infection transmission is managed during their journey through surgery even if they are anaesthetised during some of that journey.

In Table 2, I notice you use "TDF" without definition. I think table captions should be self-explanatory and would encourage defining abbreviations used in tables even if they have been defined in the text.

Phase 2 – Survey

I have quite a few reservations about this phase of the study, particularly given that it is presented as "quantitative." It is, in the end, a study of participants' perceptions, and I think

that needs to be kept front and foremost in any conclusions. Perhaps a series of questions with explanations might help convey some of my reservations.

1. What was the size of the population who were eligible to respond? In other words, what percentage responded? Was it sufficient to be meaningful? A small sample obtained randomly is useful, but this was not a random sample (you mention this yourself). There are guidelines as to what proportion of responses is reasonable for a survey, and perhaps these could be referenced.

2. Could you provide a little more detail about how the survey was piloted and perhaps validated please?

3. How have you allowed for the influence of multiple testing in your statistics? How many tests did you report?

4. More importantly, what hypotheses did you seek to test with all these statistics? I think these should be formally stated, and you probably should limit the number. Multiple testing of data sets has considerable potential to generate meaningless results. This is particularly true when making comparisons between groups. What steps have you taken to mitigate the possibility (which you acknowledge) that the decision to participate was not random and might have differed between craft groups. Thus, your results may say something about the people who responded, but do they say much about the populations from which they have been sampled?

Let me try to expand upon my concern. You say "Regarding beliefs about consequences, surgeons ($p=.037$) and nurses ($p<.001$) who unnecessarily

wore gloves were more likely to believe that gloves primarily protect the patient, and both surgeons ($p=.001$) and nurses ($p<.001$) who wore gloves unnecessarily also believed more that reducing glove use would put them at risk (these variables were not asked of anaesthetists."

1. Did you state an hypothesis about this? Is there a basis for that hypothesis? (Hypotheses should be predicated on some plausible paradigm).

2. These findings might apply to your respondents but is there any justification for suggesting that they apply to surgeons and nurses in general?

3. If you were going to test this relationship, would it not be better to test it for the entire sample first, and only look for differences between groups if you have found a significant finding for the whole population?

4. Did you do any power analyses to inform your statistics?

5. Why did you not ask anaesthetists this question? And why were anaesthetists only asked one question on self-reported unnecessary glove use. I hope this is not because you think anaesthetists don't contribute to post-surgical infection. I refer you to the work of Loftus et al and of Gargiulo et al which strongly suggests that they do. I am also surprised that

surgeons were only asked two questions, but since they tend to spend a good proportion of their time wearing sterile gloves this may be more understandable. I notice that you do not seem to have an anaesthetist in your research team and I wonder whether the infection control team who gave advice on this actually understood the role of an anaesthetist.

Similar questions pertain to; “Nurses (p=.010) and anaesthetists (p=.038)

who unnecessarily wore gloves were more likely to report that their colleagues failed to wear gloves when they should, and less likely to report that colleagues wore gloves unnecessarily. However, among surgeons, this difference did not reach statistical significance (p =.063).”

To what extent might this and other non-significant results simply represent smaller numbers for surgeons? Indeed, if you look at the individual boxes in table 5, some of the Ns are very small (e.g. 2).

More importantly, what was the effect size of the various differences? Statistical significance is of some interest, but the size of differences matters a great deal. For example, in respect of the following statement, do you think the size of difference is important?

“Experience influenced the use of non-sterile gloves, as nurses with under five years of experience reported higher unnecessary usage than those with over five years of experience (2.77 ± 0.88 vs. 2.29 ± 1.02 respectively, p=.001).”

To be clear, I am not particularly convinced that his difference is compelling.

I note that in your conclusion you say “This may explain why there was more unnecessary glove use in those with fewer than five years’ experience, as gloves appear to enhance role identity for junior OT staff.” Is this referencing the result for nurses rather than staff in general, and if so, should that be made explicit?

I may be mistaken about some or all of this, and I don’t want to go on and on about each item that you have analysed, so I have suggested that the paper requires specialist statistical review.

Discussion

In my opinion your discussion is too focused on a restatement of your results and not enough on the strengths and limitations of your work.

Conversely, I don’t think your conclusions, either in the paper or the abstract, are conclusions. They are just general summaries of what you have done and found. I think you should try to identify, justify and clearly present your conclusions. I think you need to use language that reflects the fact that your results are about the perceptions of your respondents, but I also think there are important things you could justifiably conclude.

In the main text you say, under Conclusions: “These learnings should guide the development of relevant and effective interventions, such as clearly communicating guidelines on glove

use (when to use and not to use gloves), appointing a champion to lead glove reduction efforts, fostering consistent and supportive teams within theatres,

and ensuring clear alternatives like hand gel are readily available at the point of use."

This seems to be derived from suggestions described as one of two additional themes that were not discussed in the interviews (page 22 line 12). I would have no difficulty with emphasising these suggestions in the discussion, or perhaps in a table, but if your main conclusions are drawn in reference to a minor, undiscussed part of your research what is one to think about the importance of the rest of this work? Also, in what way does your research show that these interventions would, in fact, be effective? They are good ideas, but would they work?

In your abstract, you say: "These findings contribute behavioural insights towards reducing waste in healthcare". Would it not be worth listing the two or three most important of these.

It seems to me that there are things you could conclude (these are just for illustration – you, the investigators, need to decide what you can or can't conclude, justify the conclusions and make them clear). For example, it does seem that there is a perception that sustainability is not prioritised by the NHS, and that a majority of respondents (67%) perceived that gloves were often used unnecessarily.

Two or three key conclusions of this sort would greatly strengthen the impact of your paper, and they should be included in the abstract as well as the main body of text. Also, you should perhaps try to draw conclusions from both phases of your study or even better, try to integrate the evidence from the two parts to provide stronger support for two or three key points (but see next paragraph).

Overall, you have addressed a very important topic and you have a wealth of data, much of which is very interesting. Having reached this point in reviewing your work, I am more convinced than I was at the start that you should consider splitting your report into two papers. The survey is not just an extension of your qualitative work, even though it draws from it. It has produced a substantial amount of information. Having two papers would make it easier to draw conclusions from each part. Also, I think the interview study is reasonably convincing on its own, but I have more reservations about the analysis of the survey data. I am particularly concerned that your response rate may be inadequate to justify drawing conclusions about the staff of the NHS in general, but at least you could report interesting information about the responses you did get. I would be inclined to focus on the group as a whole to maximise your sample number and emphasise that this group is contains a good mix of craft groups rather than trying to differentiate between the groups. This would reduce the number of tests and could well provide some useful insights for improving the use of NSGs.

Reviewer 2

Name Pegna, Victoria

Affiliation Kent Surrey and Sussex Deanery

Date 13-Oct-2025

COI None

Small study on single site for the interviews but an important subject and with back up of data/views using the larger questionnaire giving insight into reasons for glove usage/beliefs and views on sustainability

Reviewer 3

Name Atem, Folefac

Affiliation University of Texas Health, Biostatistics & Data Science

Date 10-Nov-2025

COI None

I do not have the necessary expertise in qualitative methods to properly assess this paper, as it falls outside my area of specialization. I recommend that we consult an expert in qualitative analysis for a more informed evaluation.

Reviewer 4

Name Zhang, Xiaofei

Affiliation Beijing Tsinghua Changgung Hospital, Department of Clinical Epidemicology and Biostatistics

Date 10-Nov-2025

COI None

The authors employed a mixed method to explore the overuse of non-sterile gloves. The suggestions for the statistics section are as follows.

Comments:

1. It would be better if there were a logic flow diagram for the mixed method and analysis.

2. Please describe briefly the specific information and related variable measurements of "a national survey, delivered on the online platform Qualtrics" in Phase 2.

3. Statistical analysis at Phase 2: the authors have explored six themes through Qualitative analysis in Phase 1. It would be clearer if the authors could provide the following relevant

information and content: a. Please provide the statistical analysis strategies and methods for each influencing factor in six themes, including Tables 4 and 5 (Variables from eight of the 14 TDF domains); b. Explain or clarify which parts of the data or contents are conducted by the ANOVA analysis method, and what the covariates are adjusted for, etc.

4. On page 17, in the Glove use behavior section: Please summarize the statistical analysis and results into a table.

VERSION 1 - AUTHOR RESPONSE

Reviewer	Reviewer comments	Author response
**3.1 Reviewer 1**	The introduction is readable, relevant, well referenced and largely to the point. I wonder whether you should include at least one sentence about the parlous state of the planet and the massive risks we currently face. Given that your paper is about gloves, which are presumably rubber or plastic, the threat they pose is presumably primarily to the Novel Entities Planetary Boundary. I think some reference to the Planetary Boundaries https://www.stockholmresilience.org/research/planetary-boundaries.html would be appropriate and perhaps a little clarification of the contribution of gloves (rather than waste more generally) on eCO2 and hence the Climate Change boundary as well as Novel Entities (notably including plastics in the oceans, and microplastics in food). This is, after all, why your study matters.	Thank you very much.<br>We have included these details in the Introduction. (p. 3):<br>“A single operation can generate up to 505.1kg of carbon dioxide equivalents (CO2e), with single-use items being one of the main contributors, threatening two areas within the Planetary Boundaries Framework, namely Novel Entities and Climate Change.”
**3.2 Reviewer 1**	I think another point that needs to be brought out a bit more in your paper, starting with the introduction, and again in the discussion, is the primary purpose of non-sterile gloves (NSGs). You do refer to this, including in your data, but I think it needs to be given more emphasis. As I understand it, these gloves (in contrast to sterile gloves) serve primarily to protect the wearer rather than patients. It is hard to see how non-sterile gloves can inhibit passing bacteria from one patient to another unless they are changed between patients, and indeed between activities. For protecting oneself, the fact that they may be less hygienic than alternatives such as alcohol gel isn’t really the only point (although it is also relevant). Is alcohol gel as protective to the clinician as NSGs? I believe that there is also some evidence worth mentioning that NSGs are often contaminated in the open boxes from which clinicians select them (my apologies - I can’t put my	We have added more detail to the Introduction and Discussion to describe when NSGs should and shouldn’t be used, and related to cross-contamination. (p.3 and 19).

	hand on the references at present). Thus, their use could actually pose a risk to patients.	
**3.3 Reviewer 1**	I wonder whether the discussion of behavioural frameworks and qualitative approaches could be more concise in the Introduction and perhaps expanded upon in the Discussion?	We have revised these sections of the Introduction to be more concise and expanded the Discussion accordingly (p. 3 and 20)
**3.4 Reviewer 1**	Your aims set the scene for two studies that are inter-related – the interviews informed the survey. This does create a rather lengthy paper with a lot of data to digest. I did find myself at least wondering whether this work should be presented as two studies, one following the other.	Thank you for this helpful comment. We carefully considered whether the interviews and survey should be presented as two separate studies. However, because the survey was directly informed by the interview findings and both components address a single overarching research question, we believe that presenting them together as a single mixed-methods study provides the most coherent account. To address concerns about length and readability, we have substantially simplified the survey results.
**3.5 Reviewer 1**	Phase 1 - Interviews<br>I found this section relatively convincing and have little to say other than that the results are very interesting. You appear to have included a reasonable number of patients for interview research, with a good representation from different craft groups. How did you decide on the number 19?	Thank you for your comment.<br>In terms of the number 19: We have now clarified the rationale for our sample size in the methods and results sections. Specifically, we used purposive sampling with predefined targets of ≥5 surgeons, ≥5 anaesthetists, and ≥5 nurses to ensure diversity of professional perspectives. Recruitment continued until thematic saturation was reached. Saturation occurred after 15 interviews overall, with subsequent interviews confirming that no new major themes were emerging. (p.4 and 7).
**3.6 Reviewer 1**	Your comment that you did not involve patients or the public because "patients are not as involved in decision making" was interesting. I suspect patients might have a view on how infection transmission is managed during their journey through surgery even if they are anaesthetised during some of that journey.	Thank you for this important point. We agree that patients may hold valuable perspectives on infection prevention and surgical safety. However, this study focused specifically on the behavioural, social and organisational influences on glove-use practices among operating theatre staff. As these decisions are made by healthcare professionals within sterile clinical environments, patient and public involvement was not included at this exploratory stage. We fully agree that future

		intervention development and evaluation should incorporate patient and public perspectives. We clarified this is the paper (p.6).
**3.7 Reviewer 1**	In Table 2, I notice you use "TDF" without definition. I think table captions should be self-explanatory and would encourage defining abbreviations used in tables even if they have been defined in the text.	Thank you, we have added the definition to Table 2.
**3.8 Reviewer 1**	Phase 2 – Survey<br>1. What was the size of the population who were eligible to respond? In other words, what percentage responded? Was it sufficient to be meaningful? A small sample obtained randomly is useful, but this was not a random sample (you mention this yourself). There are guidelines as to what proportion of responses is reasonable for a survey, and perhaps these could be referenced.	Thank you for this important question. The size of the eligible population could not be precisely determined because national workforce statistics do not identify operating theatre clinicians (surgeons, anaesthetists, theatre nurses and ODPs) as a distinct category. As recruitment was conducted via open invitations through professional organisations, hospital posters and professional networks, it was not possible to calculate a true response rate.<br><br>With 300 respondents from England, under simple random sampling assumptions this sample size would provide approximately ±6 percentage-point precision for 95% confidence intervals for estimated proportions, indicating adequate precision for descriptive analysis. However, as recruitment was voluntary and non-random, the findings are not statistically representative and are subject to self-selection bias. We have clarified this in the Methods and Limitations sections.
**3.9 Reviewer 1**	2. Could you provide a little more detail about how the survey was piloted and perhaps validated please?	We have now added more details (p.5): "[The national survey] was piloted with seven operating theatre staff (two anaesthetists, three surgeons, one theatre nurse and one ODP) to assess content validity, clarity and usability, and was revised to ensure role-specific relevance and minimise participant burden".

**3.10 Reviewer 1**	3. How have you allowed for the influence of multiple testing in your statistics? How many tests did you report?<br><br>4. More importantly, what hypotheses did you seek to test with all these statistics? I think these should be formally stated, and you probably should limit the number. Multiple testing of data sets has considerable potential to generate meaningless results. This is particularly true when making comparisons between groups.<br><br>Let me try to expand upon my concern. You say "Regarding beliefs about consequences, surgeons (p=.037) and nurses (p<.001) who unnecessarily wore gloves were more likely to believe that gloves primarily protect the patient, and both surgeons (p=.001) and nurses (p<.001) who wore gloves unnecessarily also believed more that reducing glove use would put them at risk (these variables were not asked of anaesthetists."<br><br>1. Did you state a hypothesis about this? Is there a basis for that hypothesis? (Hypotheses should be predicated on some plausible paradigm).	Thank you for this important point. This study was exploratory and theory-informed, rather than confirmatory, and we did not specify formal a priori directional hypotheses for each individual survey variable. To reduce the risk of spurious findings arising from multiple testing, we have now combined all professional groups into a single analytic group and substantially reduced the number of statistical tests reported. Continuous variables were used for constructs expected to have a greater influence on NSG behaviour (from initial qualitative findings). Therefore, statistical tests were only used for variables most expected to influence NSG use.
**3.11 Reviewer 1**	What steps have you taken to mitigate the possibility (which you acknowledge) that the decision to participate was not random and might have differed between craft groups. Thus, your results may say something about the people who responded, but do they say much about the populations from which they have been sampled?<br><br>2. These findings might apply to your respondents but is there any justification for suggesting that they apply to surgeons and nurses in general?	Thank you for this important point. Recruitment was conducted via multiple channels (professional organisations, hospital posters and professional networks) to maximise reach across different professional groups and reduce dependence on any single craft group.<br>We did provide a monetary incentive to participate in the survey which may have encouraged additional responses from people not interested in the topic. However, as participation was voluntary, we acknowledge that response patterns may have differed between professional groups and that self-selection bias cannot be fully mitigated. As such, the results should be interpreted as descriptive and hypothesis-generating rather than population-representative. Our findings were on the

		whole consistent from the interviews and survey, which does give more confidence in the generalisability of the results. A few participants were also not interested in sustainability, judging from their answer to 'I believe the operating theatre needs to become more environmentally friendly', to which 8% of respondents either were ambivalent or disagreed.
**3.12 Reviewer 1**	3. If you were going to test this relationship, would it not be better to test it for the entire sample first, and only look for differences between groups if you have found a significant finding for the whole population?	Thank you for this suggestion. We have indeed combined all participants into one group for analysis, which results in higher numbers of participants per group and therefore increased statistical strength.
**3.13 Reviewer 1**	4. Did you do any power analyses to inform your statistics?	No formal a priori power analysis was conducted, as the survey was exploratory and the total population size of UK operating theatre clinicians is not precisely known, precluding a conventional sample-size calculation. We therefore set a pragmatic target of approximately 300 respondents to achieve adequate descriptive precision, and the final sample reflects the number of responses obtained during the recruitment period.
**3.14 Reviewer 1**	5. Why did you not ask anaesthetists this question? And why were anaesthetists only asked one question on self-reported unnecessary glove use. I hope this is not because you think anaesthetists don't contribute to post-surgical infection. I refer you to the work of Loftus et al and of Gargiulo et al which strongly suggests that they do. I am also surprised that surgeons were only asked two questions, but since they tend to spend a good proportion of their time wearing sterile gloves this may be more understandable. I notice that you do not seem to have an anaesthetist in your research team and I wonder whether the infection control team who gave advice on this actually understood the role of an anaesthetist.	The decision to limit the number of glove-use items for anaesthetists and surgeons was based on feedback from the pilot study and advice from the hospital infection control team regarding role-specific relevance and participant burden, rather than any assumption about their contribution to infection risk. During piloting, several items were not perceived as directly applicable to the routine non-sterile glove practice of anaesthetists, and the survey length was reduced to minimise participant burden. We acknowledge the evidence demonstrating the role of anaesthetists in infection transmission. This rationale is now clarified in the Methods.<br><br>We do not have an anaesthetist on our research team, however we conducted survey piloting with the Association of

		Anaesthetists – several members reviewed and tested the survey and gave feedback which was implemented.
**3.15 Reviewer 1**	Similar questions pertain to; "Nurses (p=.010) and anaesthetists (p=.038) who unnecessarily wore gloves were more likely to report that their colleagues failed to wear gloves when they should, and less likely to report that colleagues wore gloves unnecessarily. However, among surgeons, this difference did not reach statistical significance (p =.063)."<br>To what extent might this and other non-significant results simply represent smaller numbers for surgeons? Indeed, if you look at the individual boxes in table 5, some of the Ns are very small (e.g. 2).	We agree that the small subgroup cell sizes substantially limit the interpretability of these comparisons and may explain some of the non-significant findings. To address this, we have now removed profession-specific subgroup analyses, combined all participants into a single analytic group, and revised the statistical reporting accordingly. This is now reflected in the Results and Methods.
**3.16 Reviewer 1**	More importantly, what was the effect size of the various differences? Statistical significance is of some interest, but the size of differences matters a great deal. For example, in respect of the following statement, do you think the size of difference is important?<br>"Experience influenced the use of non-sterile gloves, as nurses with under five years of experience reported higher unnecessary usage than those with over five years of experience (2.77 ± 0.88 vs. 2.29 ± 1.02 respectively, p=.001)."<br>To be clear, I am not particularly convinced that his difference is compelling.	We definitely agree with your point, thank you. We have re-examined the survey findings and now focus only on results that are both statistically significant and demonstrate a meaningful difference in actual numbers, to ensure relevance and interpretability.
**3.17 Reviewer 1**	I note that in your conclusion you say "This may explain why there was more unnecessary glove use in those with fewer than five years' experience, as gloves appear to enhance role identity for junior OT staff." Is this referencing the result for nurses rather than staff in general, and if so, should that be made explicit?	Thank you for noting this. We have removed this discussion point, as the profession-specific differences were small and no longer considered sufficiently distinct to support this interpretation.
**3.18 Reviewer 1**	I may be mistaken about some or all of this, and I don't want to go on and on about each item that you have analysed, so I have suggested that the paper requires specialist statistical review.	Thank you very much for the amount of feedback you have given, we really appreciate it. Based on your feedback, we have substantially revised and simplified the statistical analyses, and feel the paper is much improved.
**3.19 Reviewer 1**	In my opinion your discussion is too focused on a restatement of your results and not enough on the strengths and limitations of your work.	We have added further strengths and limitations of the paper to the Discussion section, and expanded on our results to discuss the wider context and implications (p.18-20).

**3.20 Reviewer 1**	Conversely, I don't think your conclusions, either in the paper or the abstract, are conclusions. They are just general summaries of what you have done and found. I think you should try to identify, justify and clearly present your conclusions. I think you need to use language that reflects the fact that your results are about the perceptions of your respondents, but I also think there are important things you could justifiably conclude.<br>In the main text you say, under Conclusions: "These learnings should guide the development of relevant and effective interventions, such as clearly communicating guidelines on glove use (when to use and not to use gloves), appointing a champion to lead glove reduction efforts, fostering consistent and supportive teams within theatres, and ensuring clear alternatives like hand gel are readily available at the point of use."<br>This seems to be derived from suggestions described as one of two additional themes that were not discussed in the interviews (page 22 line 12). I would have no difficulty with emphasising these suggestions in the discussion, or perhaps in a table, but if your main conclusions are drawn in reference to a minor, undiscussed part of your research what is one to think about the importance of the rest of this work? Also, in what way does your research show that these interventions would, in fact, be effective? They are good ideas, but would they work?	Thank you, we have edited the conclusions in the paper based on your recommendations.
**3.21 Reviewer 1**	In your abstract, you say: "These findings contribute behavioural insights towards reducing waste in healthcare". Would it not be worth listing the two or three most important of these.	We have edited the abstract's conclusion in light of this.
**3.22 Reviewer 1**	It seems to me that there are things you could conclude (these are just for illustration – you, the investigators, need to decide what you can or can't conclude, justify the conclusions and make them clear). For example, it does seem that there is a perception that sustainability is not prioritised by the NHS, and that a majority of respondents (67%) perceived that gloves were often used unnecessarily. Two or three key conclusions of this sort would greatly strengthen the impact of your paper, and they should be included in the abstract as well as the main body of text. Also, you should perhaps try to draw conclusions from both phases of your study or even better, try to integrate the evidence from the two parts to provide stronger support for two or three key points (but see next paragraph).	Thank you, this is very helpful. We have made significant changes to the Discussion section and Conclusions.

**3.23 Reviewer 1**	Overall, you have addressed a very important topic and you have a wealth of data, much of which is very interesting. Having reached this point in reviewing your work, I am more convinced than I was at the start that you should consider splitting your report into two papers. The survey is not just an extension of your qualitative work, even though it draws from it. It has produced a substantial amount of information. Having two papers would make it easier to draw conclusions from each part. Also, I think the interview study is reasonably convincing on its own, but I have more reservations about the analysis of the survey data. I am particularly concerned that your response rate may be inadequate to justify drawing conclusions about the staff of the NHS in general, but at least you could report interesting information about the responses you did get. I would be inclined to focus on the group as a whole to maximise your sample number and emphasise that this group is contains a good mix of craft groups rather than trying to differentiate between the groups. This would reduce the number of tests and could well provide some useful insights for improving the use of NSGs.	Thank you for this comment. We carefully considered whether to split the qualitative and survey components into two separate papers (see also our reply to 3.4). However, following your recommendations, we have substantially simplified the survey analysis, focused on the combined sample rather than profession-specific subgroups, and reduced the number of statistical tests reported in order to address your concerns about multiple testing. We now believe the survey provides complementary, hypothesis-generating insights that strengthen and extend the qualitative findings.
**4.1 Reviewer 2**	Small study on single site for the interviews but an important subject and with back up of data/views using the larger questionnaire giving insight into reasons for glove usage/beliefs and views on sustainability	Thank you very much for your comment.
**5.1 Reviewer 3**	I do not have the necessary expertise in qualitative methods to properly assess this paper, as it falls outside my area of specialization. I recommend that we consult an expert in qualitative analysis for a more informed evaluation.	-
**6.1 Reviewer 4**	1. It would be better if there were a logic flow diagram for the mixed method and analysis.	Thank you for this suggestion, we have added a diagram which should add clarity to the method and analysis (Figure 1, p.6).
**6.2 Reviewer 4**	2. Please describe briefly the specific information and related variable measurements of "a national survey, delivered on the online platform Qualtrics" in Phase 2.	Thank you for this comment. We have elaborated on this in the Methods section (p.4 and 5).
**6.3 Reviewer 4**	3. Statistical analysis at Phase 2: the authors have explored six themes through Qualitative analysis in Phase 1. It would be clearer if the authors could provide the following relevant information and content: a. Please provide the statistical analysis strategies and methods for each influencing factor in six themes, including Tables 4 and 5 (Variables from eight of the 14 TDF domains); b. Explain or clarify which parts of the data or contents are	We have not used ANOVA or adjusted for covariates. Binary variables are presented as percentages, comparing those who do- and don't use NSG appropriately; continuous variables are tested using t-tests. Then refer to the relevant text in the paper.<br>We were not able to add in the analysis section which analysis methods were used

	conducted by the ANOVA analysis method, and what the covariates are adjusted for, etc.	in each theme, as the themes were created inductively, so we could not know a priori which survey variable would be in each theme.
**6.4 Reviewer 4**	4. On page 17, in the Glove use behavior section: Please summarize the statistical analysis and results into a table.	Following the simplification of the statistical analysis, we have only one result from this section remaining, which is that '67% of participants reported using gloves unnecessarily while moving a patient when there is no visible soiling.' Therefore we think a table is no longer necessary.

VERSION 2 - REVIEW

Reviewer 4

Name Zhang, Xiaofei

Affiliation Beijing Tsinghua Changgung Hospital, Department of Clinical Epidemicology and Biostatistics

Date 23-Dec-2025

COI

The authors have revised the manuscript in response to the reviewer's comments. I have no other comments.
